# PCR on yeast colonies: an improved method for glyco-engineered *Saccharomyces cerevisiae*

**DOI:** 10.1186/1756-0500-6-201

**Published:** 2013-05-20

**Authors:** Christine Bonnet, Céline Rigaud, Emilie Chanteclaire, Claire Blandais, Emilie Tassy-Freches, Christelle Arico, Christophe Javaud

**Affiliations:** 1 Glycode S.A.S. 06 rue porte Baffat, 19140, Uzerche, France

**Keywords:** PCR, *Saccharomyces cerevisiae*, Glycosylation, Glyco-engineering

## Abstract

**Background:**

*Saccharomyces cerevisiae* is extensively used in bio-industries. However, its genetic engineering to introduce new metabolism pathways can cause unexpected phenotypic alterations. For example, humanisation of the glycosylation pathways is a high priority pharmaceutical industry goal for production of therapeutic glycoproteins in yeast. Genomic modifications can lead to several described physiological changes: biomass yields decrease, temperature sensitivity or cell wall structure modifications. We have observed that deletion of several *N*-mannosyltransferases in *Saccharomyces cerevisiae*, results in strains that can no longer be analyzed by classical PCR on yeast colonies.

**Findings:**

In order to validate our glyco-engineered *Saccharomyces cerevisiae* strains, we developed a new protocol to carry out PCR directly on genetically modified yeast colonies. A liquid culture phase, combined with the use of a Hot Start DNA polymerase, allows a 3-fold improvement of PCR efficiency. The results obtained are repeatable and independent of the targeted sequence; as such the protocol is well adapted for intensive screening applications.

**Conclusions:**

The developed protocol enables by-passing of many of the difficulties associated with PCR caused by phenotypic modifications brought about by humanisation of the glycosylation in yeast and allows rapid validation of glyco-engineered *Saccharomyces cerevisiae* cells. It has the potential to be extended to other yeast strains presenting cell wall structure modifications.

## Background

With the progress in genetic manipulations, microorganisms’ engineering for research or industrial bio-compound production is very common. Budding yeast is recognized as a GRAS (Generally Recognized as Safe) organism [1], devoid of intellectual property limitations; as such its use for industrial purposes is steadily increasing. Since the 80’s, production of biofuel by modified yeast strain is in constant development [2,3]. Yeast is also used in the manufacturing of compounds on the market of flavors or fragrances [4,5] and for the production of numerous others substances [6-9]. In 2009, *Saccharomyces cerevisiae* strains were engineered to obtain different flavonoids for use in high throughput screening of molecules in drugs discovery [10].

*Saccharomyces cerevisiae* is a particularly efficient and attractive system for the bioproduction of therapeutic proteins. Growth in neutral and well defined culture media is rapid and inexpensive as compared to higher eukaryotic systems such as CHO (Chinese Hamster Ovary) cells. Large scale production allows high yields, secretion of post-translationally modified proteins and simplified downstream purification protocols. Several recombinant therapeutic proteins have already been approved for commercialisation: Human Serum Albumin (Recombumin and Albucult, Novozymes), insulin (Actrapid, Novo Nordisk), HBV surface antigen (Pediatrix, GlaxoSmithKline), hirudine (Revasc, Aventis). Productions of other human proteins are under active studies: α-amylase [11], HPV16IL [12], immunoglobulin G [13].

Glycan residues are generally essential for mammalian glycoproteins activity [14-17]. Proteins produced in wild type yeast strains carry glycan structures radically different from native human glycoproteins; this can translate into reduced serum half-life, poor target activity and changes in their immunogenicity.

The process of *N*-glycosylation [18,19] consists in a covalent linkage of a specific oligosaccharide on a nascent protein in the endoplasmic reticulum. This first step is conserved through all Eukaryotes. The maturation process occurs in the Golgi apparatus and leads to polymannosylated, complex or hybrid structures, and determines differences between species. In *Saccharomyces cerevisiae* strains, a set of mannosyltranferases will add mannose residues to the core glycan until the protein is hypermannosylated; in mammals the glycan structures are more complex, composed of N-acetylglucosamine, galactose, sialic acid or fucose. The engineering of the yeast *N*-glycosylation pathway allows therapeutic glycoproteins production bearing homogeneous human type glycan structures. This homogeneity increases protein therapeutic efficiency and reproducibility between production batches [20].

Glycode has developed technologies to enhance recombinant glycoprotein production through selective modification of glycosylation in yeast *Saccharomyces cerevisiae*. In the GlycodExpress™ technology (patent WO/2008/095797), *Saccharomyces cerevisiae* has been modified by sequential deletion of mannosyltransferases and glycosyltransferases expression cassettes introduction into the yeast genome by homologous recombination. This offers several advantages: strain stability with time, full tracking of genotypic and phenotypic changes and possibility to reengineer the strain identically in case of spontaneous recombination. For the YAC-Express™ approach (patent WO/2012/013823), *Saccharomyces cerevisiae* has been engineered by introduction of a YAC (Yeast Artificial Chromosome) containing a cluster of glycosyltransferases expression cassettes into a yeast strain deleted for mannosyltransferases. Among the numerous advantages of the YAC-Express™ technology is the absence of direct chromosomal modification, short time-frame for engineering a new strains and extension of the YAC technology to a wide variety of strains. The first essential step to humanise yeast *N*-glycosylation is the deletion of sequences encoding specific mannosyltransferases, such as OCH1 and MNN1, leading to strains producing glycoproteins with a core Man_8_GlcNAc_2_ type structure [21-24].

The quality of these initial glyco-engineered strains is guaranteed by a rigorous validation process. The first step is a PCR (Polymerase Chain Reaction) on the yeast genome to check the presence of the deletion cassette and the absence of the targeted mannosyltransferase sequence. Since the early 90’s, several techniques have been described to carry out DNA amplification directly on yeast colonies [25]. A protocol based on classical PCR methods [26] has been adapted for the identification of pathogenic fungi [27]. The use of Zymolyase or lyticase is reported to allow a better disruption of the cell wall and, consequently, a better amplification [28]. During our validation of modified strains it appeared that *N*-glycosylation modifications prevented PCR amplification directly on yeast colonies. To circumvent this we have developed a new protocol based on a micro-plate culture phase on a *Δoch1Δmnn1* strain (YiMMOgène) derived from the BY4742 laboratory strain. Culture duration and location of the targeted sequence have no impact on the efficiency of this method.

## Findings

### Classical protocols for PCR on yeast colonies showed low amplification efficiency on glyco-engineered strains

The wild type laboratory strain BY4742 and the modified *Δoch1Δmnn1* YiMMOgène strain were restreaked on YPD (Yeast Peptone Dextrose) plates (47 colonies for each strain). The YiMMOgène strain deficient in mannosyltransferase activity has growth defects; it needs several days for colonies to appear on agar plate culture. After 5 days of growth at 30°C, each colony was tested for the amplification of 900 bp (base pair) of MNN5 sequence. This gene is located on chromosome X and encodes a Golgi mannosyltransferase responsible for the linkage of an α-1,2-mannose on the glycan outer chain [29]. Two different protocols using two different DNA polymerases have been successively carried out. Amplification or PCR efficiency was calculated as follows: efficiency= (number of amplifications obtained/number of colonies tested) *100.

**Figure 1:**
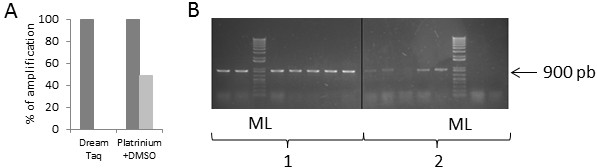
**Efficiency of MNN5 gene amplification from wild type and modified strains. A**) Percentage of colonies picked from Petri dishes that amplified MNN5. The experiment was carried out on 47 colonies for each strain. Dark grey: BY4742; light grey: YiMMOgène. Amplification was carried out using the DreamTaq or the Platinium Taq. **B**). PCR product visualisation, after amplification with the Platinium Taq, on a 1% agarose gel stained with SYBR safe. Left panel (1): BY4742, right panel (2): YiMMOgène, ML: Molecular ladder.

First, PCR was carried out with a widely used DNA polymerase (Dream Taq, Fermentas). All BY4742 colonies showed MNN5 amplifications, whereas no amplification was observed on YiMMOgène colonies (Figure 1A). Secondly, a Hot Start DNA polymerase (Platinium Taq, Invitrogen) was used in presence of DMSO (Dimethylsulfoxide) in the reaction mix. DMSO is known to inhibit secondary structures and to promote access to DNA [30,31]. The percentage of amplification was of 100% for BY4742 and 49% on YiMMOgène (Figure 1A). The different PCR products were assessed by agarose gel electrophoresis. In addition to poor amplification efficiency, amplifications from YiMMOgène colonies presented low quality and irregular intensities (Figure 1B). The use of different DNA polymerases [classical (DyNAzyme, Finnzymes), high fidelity (Taq-Phusion, New England Biolabs or Taq Isis, Qbiogene) or specific for long fragment (Taq long-expand, Roche)], under several PCR conditions (extension time, denaturation temperature, MgCl_2_ concentration) and addition of various concentrations of secondary structure inhibitors (DMSO, formamide) did not increase amplification efficiency beyond 50% (unpublished observations).

Since the importance of annealing temperature has been clearly demonstrated [32,33], experiments were conducted varying this parameter. Temperatures from 53°C to 60°C were tested, in combination with different primer pairs; however, this did not improve amplification efficiency or quality significantly (unpublished observations).

The initial hypothesis was that the low PCR efficiency on engineered *Saccharomyces cerevisiae* strains was due to complex DNA structure. However, variation in all different parameters (different Taq polymerases, PCR programs, inhibitors of secondary DNA structure) did not afford improvement in PCR product quantity or quality.

### A lyticase treatment can increase PCR efficiency

Yeast cells are encapsulated by a rigid but dynamic cell wall structure. It determines the shape of the organism; it provides osmotic protection and forms a physical barrier to the extracellular environment [34,35]. Breaking this organelle is a prerequisite for DNA amplification by PCR on yeast colonies. Different techniques, applied to DNA or protein extraction, have been described for several decades: lysis by chemicals such as SDS (Sodium Dodecyl Sulfate) [36,37], cell wall disruption by thermal choc [38-40], sonication [41,42] and microwaves [43,44]. Experiments based on these methods were successively carried out: they did not improve PCR efficiency (unpublished observations).

**Figure 2:**
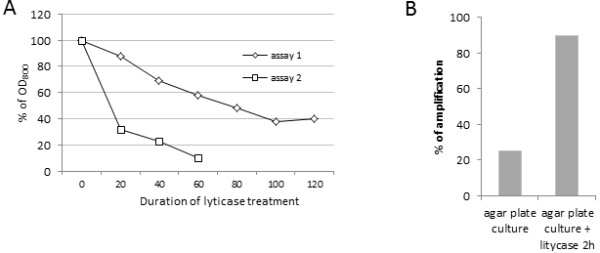
**Lyticase treatment of the YiMMOgène colonies can improve PCR efficiency. A**) OD_800_ measurement during lyticase treatment, 2 independent assays. OD_800_ was measured on an Ultrospec 2100pro (Amersham). **B**) Percentage of MNN5 amplification from 20 colonies picked on Petri dishes treated or untreated with lyticase for 2 h.

Enzymatic permeabilisation with zymolyase or lyticase is very common [45,46]. Lyticase hydrolyzes linear glucose polymers at beta-1,3-linkages and is the preferred enzyme to digest cell walls and generate spheroplasts from *Saccharomyces cerevisiae* strains. When a OD_800_ decrease of 80% is observed in the reaction system, the yeast cells can be considered as completely lysed. We submitted engineered colonies to a classical 20 minute lyticase treatment but did not succeed in genomic DNA amplification (unpublished observations). To check the sensibility of YiMMOgène to enzymatic lysis, we performed 2 independent assays on fresh yeast cells collected from 5 days agar plate culture. The initial OD_800_ in the reaction mix were 2,2 and 1,6 respectively. The OD_800_ was verified every 20 minutes (Figure 2A). It appears that the permeabilisation efficiency is not reproducible.

In a second assay, 20 YiMMOgène colonies were picked from an agar plate culture and treated with lyticase for 2 h. After centrifugation, spheroplasts were picked and submitted to amplification. The same 20 colonies were also assayed for PCR directly from the agar plate. The lyticase treatment increases PCR efficiency from 25% to 90% (Figure 2B). However, lyticase permeabilisation appears to be random and the procedure time consuming and too expensive for use in high throughput screening.

These experiments demonstrate that alteration of YiMMOgène cell wall properties could be an explanation for low PCR efficiency on agar plate colonies.

### A microculture phase allowed good amplification on yeast altered for *N*-glycosylation

PCR is performed on 5 day old colonies; at this point yeast has reached the early stationary growth phase during which the cell wall becomes resistant as compared to exponential growth phase (thinner cell wall). Using cells from a liquid culture in exponential phase may help to improve the PCR reaction. However, in order to screen numerous yeast colonies, it is important to conserve a limited volume of culture.

**Figure 3:**
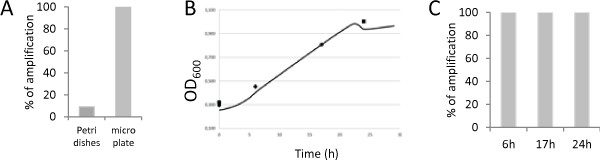
**Efficiency of MNN5 gene amplification from liquid culture of modified strain. A**) Percentage of MNN5 amplification from 47 colonies picked on Petri dishes and 47 colonies picked from liquid cultures in micro-plates. **B**) Growth curve in micro-plate (YPD medium), average of two colonies of YiMMOgène. The OD_600_ was measured in a micro-plate reader. ♦: OD_600_ after 6 hour culture (early exponential phase). ●: OD_600_ after 17 hour culture (late exponential phase). ▄: OD_600_ after 24 hour culture (early stationary phase). **C**) Percentage amplification of MNN5 from 47 YiMMOgène after different culture times in micro-plate. All amplifications have been carried out with the Platinium Taq.

YiMMOgène strain was restreaked on YPD plates. After 5 days at 30°C, 47 colonies were diluted in 50 μL of YPD on a micro-plate and grown at 30°C, under agitation, for 18 hours. The cells were then collected directly, either from Petri dishes or from liquid cultures. PCR was carried out on the MNN5 sequence with the Platinium Taq DNA polymerase in presence of DMSO. Only 9% amplification efficiency was observed on colonies from YPD plate; however 100% was obtained after a liquid culture phase (Figure 3A). This confirms the importance of yeast culture conditions (solid vs. liquid medium).

In a subsequent assay we determined the effect of culture duration on PCR efficiency. A growth curve in micro-plate was carried out on two distinct YiMMOgène colonies (Figure 3B). Amplifications of 47 liquid cultures were performed from cells in early exponential phase (6 h), late exponential phase (17 h) or early stationary phase (24 h). As shown in Figure 3C, there is no difference in PCR efficiency correlated to a particular time of culture, in the range tested.

### The efficiency of the developed method was repeatable

**Figure 4:**
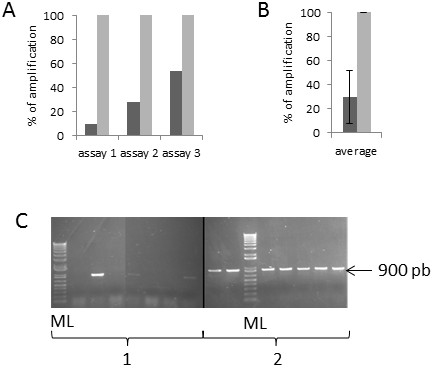
**MNN5 gene amplification efficiency on modified strain colonies picked from Petri dishes or micro-plate cultures. A**) 3 independent assays carried out on 47 colonies for each condition. Dark grey: Petri dishes cultures, light grey: micro-plate cultures. **B**) Average of the 3 assays presented in A (standard deviation =22.07). C) PCR product visualised on a 1% agarose gel stained with SYBR safe. Left panel (1): Petri dishes, right panel (2): micro-plate cultures, ML: Molecular ladder. All amplifications were carried out using Platinium Taq.

In addition to the previous assay, two distinct tests targeting the MNN5 sequence were conducted on 47 YiMMOgène colonies, picked from a Petri dish after 5 days of growth or from a micro-plate after 6h of liquid culture. An efficiency of 100% was observed from liquid culture, while it varied from 9% to 53% (average of 30%) from agar plate cultures (Figure 4). A great improvement in amplification quality was observed (Figure 4C).

### The efficiency of the developed method was not target sequence dependent

**Figure 5:**
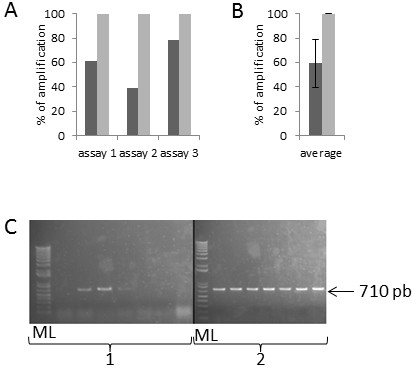
**MNN2 gene amplification efficiency on modified strain colonies picked from Petri dishes or micro-plate cultures. A**) 3 independent assays carried out on 23 colonies for each condition. Dark grey: Petri dishes cultures, light grey: liquid cultures. **B**) Average of the 3 assays presented in A (standard deviation=19.55). **C**) PCR product visualized on a 1% agarose gel stained with SYBR safe. Left panel (1): Petri dishes, right panel (2): micro-plate cultures, ML: Molecular ladder. All amplifications have been carried out with the Platinium Taq.

Three distinct assays were carried out for the amplification of 710 pb of the MNN2 sequence. MNN2 is located on the chromosome II and encodes a golgi α-1,2-mannosyltransferase [29]. Mnn5p and Mnn2p have similar functions however their sequences are different, with only 49.9% homology. Moreover, the chosen primers pairs were located in different regions of the ORF (Open Reading Frame) targeted. The methodology used for MNN5 was applied to MNN2 gene amplification on 23 colonies. As shown in Figure 5, percentage amplification from agar plate cultures was higher but still insufficient with an average of 59% (from 39% to 78%), whereas the percentage obtained after a liquid culture was constant at 100%. Again, the quality of the PCR product is improved using a liquid culture step (Figure 5C).

## Discussion

*Saccharomyces cerevisiae* cells deleted for several *N*-mannosyltransferase sequences appear to be “resistant” to yeast PCR protocols.

In the classical method, amplification is carried out on colonies directly picked from agar plate culture; in the new approach presented here, colonies are submitted to a short liquid culture phase before being analyzed. This additional step allows a significant improvement of PCR efficiency, from 30% to 100%, on a *Δoch1Δmnn1* strain. For the PCR definition of efficiency (percentage of amplification obtained) only the quantitative aspect, and not the qualitative aspect (intensity of amplification), is considered. Indeed, while performing screening, we are looking for a positive or negative result; the quality of amplification, even if we have noted an improvement, is not so important in this specific application.

Various parameters concerning the PCR (annealing temperature, denaturation time…), the reaction buffer composition (MgCl_2_ concentration, specific GC buffer…) and the addition of DNA secondary structure inhibitors (DMSO…) have been tested, but none of these provided a sufficient increase in PCR efficiency. This suggests that difficulty in PCR amplification was due to alteration in yeast cell properties rather than to DNA secondary structure modifications.

Amplification protocols have been carried out on 5 day colonies. Clones are invisible on the plate until the 5th day due to the disturbed growth of OCH1 deleted strains; at this culture time cells have just reached the early stationary phase and PCR efficiency is very low. In contrast, after 24 h of liquid culture YiMMOgène strain is also in the early stationary phase but the PCR efficiency reached 100%. Moreover, when 5 day old wild type colonies are analyzed PCR efficiency is of 100%. The amplification failure is not dependent of the aged of the colony but rather on the effect of glyco-engineering.

Differences in PCR efficiency have been observed between wild type and modified strains, but also in modified yeast, between agar and liquid culture. In *Saccharomyces cerevisiae* the cell wall represents around 30% of the cell dry weight and is largely composed of neutral polysaccharides for the inner layer (85%) and heavily glycosylated mannoproteins (15%) for the outer layer [47,48]. Depending on growth conditions at least 20 different mannoproteins, are present and they likely have specific functions. These glycoproteins, particularly their *N*-linked carbohydrate side-chains, influence the cell wall permeability to macromolecules [49-52]. It has been reported that alteration of glycosylation, by deletion or mutation of mannosyltransferases, drastically increases cell wall porosity [53,54] and chitin synthesis [55]. Interactions between “cell wall stress”, glycosylation and chitin deposit in the cell wall have been already described [56-58]. Although accounting for only 1–2% of the wild type cell wall under vegetative growth, chitin can contribute up to 20% of this structure under cell wall stress conditions [54]. As its crystalline structure confers resistance to the cell wall, an increased amount of chitin can dramatically raise the cell’s resistance to chemical or mechanical disruption, impairing the capacity to access the DNA matrix and to perform PCR on yeast colonies. Numerous methods have been described to break or permeabilise yeast cell wall. We tested enzymatic lysis: the PCR results show that disturbing the cell wall allows an increase of efficiency by more than 3 fold. However, because of the random results of permeabilisation, the time needed to perform the experiment and the cost of the enzyme, lyticase procedure is not suitable for the screening of several hundreds of colonies. Stationary-phase cells are physiologically, biochemically, and morphologically distinct from exponentially growing cells. Polysaccharide composition, structure and thickness of the cell wall are tightly controlled and vary considerably, depending on environmental conditions [59,60]. The major change concerns the large size mannoprotein material, mainly because of variations in the amount of *N*-glycan linked mannose residues [61]. Cell wall porosity of batch-grown *Saccharomyces cerevisiae* is maximal in the early exponential phase and decreases rapidly to lower levels in later stationary growth phases [50]. In the improved protocol, cells are analyzed during the exponential growth phase; unlike those from agar plate cultures which are in early stationary phase.

It has been shown that the *mnn1och1* mutants tend to aggregate, probably because of glycosylation defects [53] and this was observed with YiMMOgène strain. Aggregation limits access to numerous cells and so to nuclear DNA thus reducing efficiency of direct amplification on yeast colonies from agar plate cultures.

To conclude, the increase of chitin deposit in the cell wall of engineered yeasts, the structure of this organelle in early stationary phase and cell aggregation in *Δoch1Δmnn1* yeast strains can each partially explain the loss of PCR efficiency on yeast colonies. However, it is likely that the convergence of the three phenomena leads to the absence of amplification during PCR process on YiMMOgène strain colonies. The improved protocol developed in this work by-passes those difficulties and it has the potential to be applied to yeast strains presenting cell wall structure modifications.

## Conclusion

Characterisation of genetically engineered cell lines is essential before they can be released on the market. PCR is usually an efficient tool to analyze genomes. Unfortunately, DNA sequence modifications can lead to phenotypic changes that may render genomes inaccessible for such studies. An improved method for PCR on yeast colonies has been developed for validation of humanised *Saccharomyces cerevisiae* strains. This protocol can be easily implement in yeast labs and does not need any specific instrumentation. It allows the rapid screening, in a cost effective and reproducible manner, of several hundreds of yeast colonies in parallel.

## Methods

### Yeast strains

BY4742 (*MATalpha, his3delta1, leu2delta0, lys2delta0, ura3delta0*) is a laboratory *Saccharomyces cerevisiae* strain, derived from the S288C strain. It was obtained from ATCC (#201389). The YiMMOgène strain (*MATalpha, his3delta1, leu2delta0, lys2delta0, ura3delta0, och1Δ::* KanMX4, *Mnn1Δ*:: Hph) was constructed at Glycode S.A.S., it is derived from the BY4742 strain. It was engineered by a PCR-based gene deletion strategy [62] that allows the deletion of the ORF of interest from the yeast genome. The OCH1 gene was replaced with the KanMX cassette that confers resistance to kanamycine. The MNN1 gene was replaced with a Hph module that confers resistance to hygromycine.

### Media and culture conditions

YPD (Yeast Peptone Dextrose) complete medium was purchased from Formedium. The liquid medium was prepared as recommended by the supplier; for solid medium, 20 g/L agar from Formedium was added to the mixture before autoclaving. Culture micro-plates were purchased from Greiner Bio-One (96 well, round bottom, well volume 250 μL). Petri dishes were purchased from Greiner Bio-One (100 mm).

A frozen 80% glycerol stock containing the yeast strain was streaked onto an YPD agar plate. The plate was incubated at 30°C, until individual colonies appeared. 47 of those colonies were isolated onto YPD agar plates and incubated 5 days at 30°C. Each yeast colony was seeded, with a toothpick, into 50 μL YPD liquid medium in micro-plate well and incubated at 30°C, under agitation (200 rpm), during a pre-determinate time (6 h, 17 h or 24 h). The OD_600_ was measured by a micro-plate reader (Infinite® 200 PRO from Tecan).

### Determination of the optimal annealing temperature

Primers used were purchased from Eurofins MWG Operon (Table 1). The T_m_ was initially calculated using the (4(G+C) + 2(A+T)) formula. For PCR on liquid culture colonies, different annealing temperatures surrounding the theoretical number were tested and the optimal T_m_ found for both primer pairs was 58°C.

**Table 1:** Primers used in this study

**Targeted gene**	**Primer name**	**Primer sequence**	**T**_**m**_	**Product lenght**
MNN5	CR067-Fw	5′-ACGACTGGTTCCTAGCACAAC-3′	64°C	900 bp
	CR068-Rv	5′AAAAGGCGGGAGGGGTGAC-3′	62°C	
MNN2	CR064-Fw	5′-TTCCTCAGACGCCGCCAG-3	60°C	710 bp
	CR064-Rv	5′GGCCACATGACCAAGCCC-3′	60°C	

Fw: forward, Rv: reverse.

### PCR amplification

With the DyNAzyme DNA polymerase (Finnzymes) PCR was performed in 25 μL final volume, with the 2X supplier mix that contains both the buffer and the enzyme. Primers were added to a final concentration of 0.5 μM and H_2_O up to 25 μL.

With the Platinium Taq DNA polymerase (Invitrogen), the PCR mix was performed with final concentrations of: 1x manufacturer-supplied buffer, 1.5 mM MgCl_2_, 0.2 mM dNTPs, 0.3 mM primers, 2.4% DMSO, 0.5 U of the Hot Start enzyme, H_2_O to 20 μL. The PCR was set up with yeast cells picked from liquid culture in micro-plate or agar plate culture. The reactions were performed on an Eppendorf Thermal cycler mastercycler ep gradient. Primers used are presented in Table 1. Thermocycling conditions are presented in Table 2. After cycling, 7.5 μL of each sample were loaded on a 1% agarose gel stained with SYBR safe for visualization.

**Table 2:** Thermocycling conditions used in this study

Initial denaturation	95°C	15 min	1 cycle
Denaturation	95°C	30 s	40 cycles
Annealing	58°C	30 s	
Extension	72°C	1 min	
Final extension	72°C	5 min	1 cycle

### Lyticase treatment

Yeast from a 5 days agar plate culture were diluted in 5 ml H_2_O, at approximately 1.5<OD_600_<2.5. After centrifugation for 5 minutes at 2000 g, the pellet was incubated in 750 μl of pretreatment buffer (100 mM Tris HCl pH 9.4, 1 mM DTT) for 10 minutes at 30°C and centrifuged for 5 minutes at 1000 g. The supernatant was discarded and the pellet carefully resuspended in 500 μl of spheroplast buffer (0.6 mM sorbitol, 50 mM Tris pH 7.4, 1 mM DTT). 400 U of lyticase in H_2_O was added and the reaction performed at 30°C, under gentle shaking for 120 minutes. Evolution of the reaction is verified by measuring at OD_800_: reaction is complete when the OD decreased from 100% to 20%.

For the treatment before PCR reaction, yeast colonies have been picked from a 5 days agar plate culture and diluted to H_2_O to around 1 OD_600_. The reaction was carried out as describe above, without following the OD_800_. After 2 h, spheroplasts were harvested by centrifugation and collected with a toothpick; PCR was performed as described in 4th paragraph of the methods section.

## Abbreviations

PCR: Polymerase chain reaction; GRAS: Generally recognized as safe; CHO: Chinese hamster ovary; YAC: Yeast artificial chromosome; YPD: Yeast peptone dextrose; DMSO: Dimethylsulfoxide; SDS: Sodium dodecyl sulfate; bp: base pair; ORF: Open reading frame; DTT: DL-dithiothreitol; OD: Optical density.

## Competing interests

The authors declare no competing interests.

## Authors’ contributions

CB and CR designed the study. EC, CB and ETF participated to the design reflexion. EC performed the experiments. CB, CR, ETF, CA and CJ contributed to results analysis. All authors contributed to the manuscript and have read and approved the final manuscript.
